# Supplementary material for: Dynamic transcriptomic profiles of zebrafish gills in response to zinc supplementation
Source: BMC Genomics. 2010 Oct 11;11:553. doi: 10.1186/1471-2164-11-553 (PMC3091702; doi:10.1186/1471-2164-11-553)
Supplement: Additional file 2 — Interactive Direct Interaction Network representing the molecular interactions between zinc, copper, iron, calcium and proteins encoded by transcripts changed by zinc supplementation. Mini web-site containing index.html and hyperlinked pages in subdirectory describing a Direct Interaction Network automatically generated based on curated interactions contained within the proprietary PathwayArchitect database. Ovals represent proteins and the circles symbolize metal ions. Objects are coloured by their abundance in zebrafish at the time-point they were significantly different from the control is a scale from -4 fold (dark green) to +4 fold (dark red). Where significant differences were found at more than one time-point, the colour overlay shows expression at the first instance. Dark blue squares denote 'binding', and light blue squares 'expression'; green squares stand for 'regulation', green diamonds for 'metabolism', and green circles for 'promoter binding'. Arrow heads indicate directionality of the interaction where annotated. All nodes and edges can be further interrogated by selecting the relative area of the image. [file 1471-2164-11-553-S2.zip › PathwayArchitect Zn xs DIN/484740.html]

# REGULATION:

|  |  |
| --- | --- |
| Type | REGULATION |
| Effect | Positive |


---

|  |  |
| --- | --- |
| Score | 0 |


---

|  |  |
| --- | --- |
| Reference Count | 16 |


---

|  |  |
| --- | --- |
| Mechanism | Unknown |


---

|  |  |
| --- | --- |
| Reference:0 || Sentence | "In conclusion: 1) activation of Ca2+ influx by formyl-methionyl-leucyl-phenylalanine depends on the elevation of [Ca2+]i, the latter being initiated by Ca2+ mobilization from intracellular stores; 2) Ins(1,3, 4,5)P4 is a strong candidate for maintaining receptor-mediated activation of Ca2+ influx in differentiated HL-60 cells." |
| PMID | 2540183 |
| Year | 1989 |
| Species | Human |
| Journal | J Biol Chem |
| RefScore | 2 |
| Source | PArchNLP |
  |
|  |  |


---

|  |  |
| --- | --- |
 Reference:1 || Sentence | "Only those inositol trisphosphates able to evoke Ca2+ release from internal stores can support the action of Ins 1,3,4,5 P4 in evoking responses that are acutely dependent on extracellular Ca2+ (Ca2+ influx)." |
| PMID | 2670241 |
| Year | 1989 |
| Species | Human |
| Journal | Cell Calcium |
| RefScore | 2 |
| Source | PArchNLP |
  ||  |  |


---

|  |  |
| --- | --- |
 Reference:2 || Sentence | "Such an enzyme activity, if present in hormonally sensitive cells, could provide a mechanism for maintaining constant concentrations of Ins(1,4,5)P3 and Ins(1,3,4,5)P4, important for stimulation of Ca2+ entry after Ca2+ mobilization." |
| PMID | 3261978 |
| Year | 1988 |
| Species | Human |
| Journal | Biochem J |
| RefScore | 2 |
| Source | PArchNLP |
  ||  |  |


---

|  |  |
| --- | --- |
 Reference:3 || Sentence | "The activity of the Ins-1,4,5-P3 kinase obtained from either resting or stimulated Jurkat cells was enhanced in vitro by increasing the concentration of free Ca2+ from 0.1 to 0.5 microM." |
| PMID | 3494750 |
| Year | 1987 |
| Species | Human |
| Journal | J Clin Invest |
| RefScore | 1 |
| Source | PArchNLP |
  ||  |  |


---

|  |  |
| --- | --- |
 Reference:4 || Sentence | "Both Ins(1,3,4,5)P4- and Ins(1,4,5)P3-induced Ca2+ mobilizations were heparin sensitive." |
| PMID | 8232232 |
| Year | 1993 |
| Species | Human |
| Journal | Mol Pharmacol |
| RefScore | 2 |
| Source | PArchNLP |
  ||  |  |


---

|  |  |
| --- | --- |
 Reference:5 || Sentence | "Generation of Ins(1,4,5)P3 may mobilize Ca2+ from the space between the nuclear membranes and thus increase nucleoplasmic Ca2+." |
| PMID | 12237449 |
| Year | 2002 |
| Species | Human |
| Journal | Sci STKE |
| RefScore | 1 |
| Source | PArchNLP |
  ||  |  |


---

|  |  |
| --- | --- |
 Reference:6 || Sentence | "However, although microinjection of D-myo-inositol 1,4,5-trisphosphate [Ins(1,4,5)P3] into individual smooth muscle cells always produced significant increases in intracellular calcium levels in the injected cell, the intercellular spread of Ca2+ in response to Ins(1,4,5)P3 was more variable than for Ca2+ injections." |
| PMID | 1325115 |
| Year | 1992 |
| Species | Human |
| Journal | Am J Physiol |
| RefScore | 2 |
| Source | PArchNLP |
  ||  |  |


---

|  |  |
| --- | --- |
 Reference:7 || Sentence | "We suggest that Ins(1,4,5)P3 acts as the second messenger mediating transmembrane Ca2+ influx through specific Ca2+-permeable channels in mitogen-stimulated T-cell activation." |
| PMID | 2434867 |
| Year | 1987 |
| Species | Human |
| Journal | Nature |
| RefScore | 1 |
| Source | PArchNLP |
  ||  |  |


---

|  |  |
| --- | --- |
 Reference:8 || Sentence | "Blockade of Ca2+ entry into the cells by Ni2+, or incubation with EGTA, which reduces agonist-stimulated accumulation of Ins(1,4,5)P3, had no effect on the Li(+)-dependent increase in Ins(1,4,5)P3." |
| PMID | 7575458 |
| Year | 1995 |
| Species | Human |
| Journal | Biochem J |
| RefScore | 1 |
| Source | PArchNLP |
  ||  |  |


---

|  |  |
| --- | --- |
 Reference:9 || Sentence | "The intracellular receptor that mediates Ins(1,4,5)P3-stimulated Ca2+ mobilization has been purified and functionally reconstituted, and its amino acid sequence deduced from its cDNA sequence." |
| PMID | 1663251 |
| Year | 1991 |
| Species | Human |
| Journal | Pharmacol Ther |
| RefScore | 0 |
| Source | PArchNLP |
  ||  |  |


---

|  |  |
| --- | --- |
 Reference:10 || Sentence | "D-Ins(1,4,5)P3-induced mobilization of Ca2+ was transient, and Ca2+ reuptake followed D-Ins(1,4,5)P3 metabolism closely." |
| PMID | 1646949 |
| Year | 1991 |
| Species | Human |
| Journal | Mol Pharmacol |
| RefScore | 1 |
| Source | PArchNLP |
  ||  |  |


---

|  |  |
| --- | --- |
 Reference:11 || Sentence | "The involvement of inositol lipid metabolism in agonist-mediated Ca2+ signaling by Ins 1,4,5-P3 has become firmly established." |
| PMID | 2496641 |
| Year | 1989 |
| Species | Human |
| Journal | Annu Rev Physiol |
| RefScore | 0 |
| Source | PArchNLP |
  ||  |  |


---

|  |  |
| --- | --- |
 Reference:12 || Sentence | "Overwhelming evidence supports the concept that Ins 1,4,5-P3, the product of PIP2 hydrolysis, is responsible for the initial agonist-induced Ca2+ transient by mobilization of Ca2+ from a specialized intracellular store." |
| PMID | 2190806 |
| Year | 1990 |
| Species | Human |
| Journal | Environ Health Perspect |
| RefScore | 2 |
| Source | PArchNLP |
  ||  |  |


---

|  |  |
| --- | --- |
 Reference:13 || Sentence | "These findings suggest that intracellular Ca2+ signals activated via stimulation of Ins(1,4,5)P3 receptor are involved in the release of ATP evoked by Ang II." |
| PMID | 12054523 |
| Year | 2002 |
| Species | Human |
| Journal | Biochem Biophys Res Commun |
| RefScore | 1 |
| Source | PArchNLP |
  ||  |  |


---

|  |  |
| --- | --- |
 Reference:14 || Sentence | "CIF, which stimulates Ca2+ entry directly on microinjection, was active at dilutions at which it had no direct effect by augmenting both initial rapid Ins(1,4,5)P3-mediated Ca2+ discharge-activated currents and later sustained Ca2+ entry-activated currents." |
| PMID | 8809059 |
| Year | 1996 |
| Species | Human |
| Journal | Biochem J |
| RefScore | 2 |
| Source | PArchNLP |
  ||  |  |


---

|  |  |
| --- | --- |
 Reference:15 || Sentence | "These data show a close temporal and dose-response relationship between Ca2+ entry via L-type voltage-sensitive Ca2+ channels and Ins(1,4,5)P3 formation following depolarization with K+, indicating that Ca2+ influx can activate phospholipase C in SH-SY5Y cells." |
| PMID | 8528562 |
| Year | 1995 |
| Species | Human |
| Journal | Br J Pharmacol |
| RefScore | 1 |
| Source | PArchNLP |
  |


---

|  |  |
| --- | --- |
